# Supplementary material for: Dual-Responsive and Reusable Optical Sensors Based on 2,3-Diaminoquinoxalines for Acidity Measurements in Low-pH Aqueous Solutions
Source: Sensors (Basel). 2023 Mar 9;23(6):2978. doi: 10.3390/s23062978 (PMC10057592; doi:10.3390/s23062978)
Supplement: Supplementary file 1 [file sensors-23-02978-s001.zip › sensors-2254358-supplementary.pdf]

Supporting Information

Table of Contents:

1. **Synthesis of compounds QC1 and QC8.** 2
2. **NMR spectra for compounds** 9
3. **HRMS (ESI) spectra for compounds** 14
4. **Spectroscopic studies of QC1 and QC8 in solutions** 19
5. **Monolayer of QC8 at the air–water interface** 21
6. **LB and LS films of QC8** 22
7. **Halochromic materials for qualitative pH measurement in the range of 1–4** 24

1. Synthesis of compounds QC1 and QC8.

1.1. General considerations

Unless otherwise noted, all chemicals and starting materials were obtained commercially from Acros® or Aldrich® and used without further purification. Cs_2CO_3 was dried under reduced pressure at 120 °C for 48 h. Chloroform (analytical grade) for physicochemical studies and methanol (99.8%) were purchased from Merck. Deionized water (18.2 MΩ cm, pH ~5.5) was produced by a Vodoley cartridge purificator (SPE Himelektronika, Russia). All metal salts used were perchlorates of general $M(ClO_4)n{\bullet}xH_2O$ formula. *Caution!* Although no problems were experienced, perchlorate salts are potentially explosive when combined with organic ligands and should be manipulated with care and used only in very small quantities. DMA was dried with molecular sieves.

^1H and ^{13}C spectra were acquired either on a Bruker Avance III 500 MHz spectrometers. Chemical shifts are expressed in parts per million (ppm), referenced on the δ scale by using residual non-deuterated solvent signals as internal standard for ^1H and ^{13}C NMR spectroscopies. The coupling constants are expressed in units of frequency (Hz). MALDI-TOF mass-spectra were obtained on a Bruker Ultraflex II LRF 2000 mass-spectrometer in positive ion mode with dithranol matrix. Accurate mass measurements (HRMS) were made on a THERMO LTQ Orbitrap XL equipped with an electrospray ionization (ESI) source in positive mode unless otherwise stated. Solutions in $CHCl_3$/methanol (1:1, v/v) were used for the analysis. IR spectra were registered on Bruker Vector 22 spectrophotometer. Universal micro-ATR sampling accessory (Pike) was used in order to obtain IR spectra of solid samples. Analytical thin-layer chromatography (TLC) was carried out using Merck silica gel 60 F-254 plates (precoated sheets, 0.2 mm thick, with fluorescence indicator F254).

All the spectrometers were available at the "Pôle Chimie Moléculaire", the technological platform for chemical analysis and molecular synthesis (http://www.wpcm.fr) which relies

on the Institute of the Molecular Chemistry of University of Burgundy and WelienceTM, a Burgundy University private subsidiary.

pH measurements were carried out using portative Ecotest 2000 pH-meter with combined ESK-10601/7 glass electrode. The electrode was calibrated with commercial buffers (pH = 4.01, 6.86 and 9.18). Protonation studies for solutions were conducted in a glass beaker, equipped with a magnetic stirrer and the pH-electrode. For studies of halochromic properties of Langmuir monolayers, polymer films, test-strips and LB/LS films series of aqueous solutions with different pH were prepared by adding aliquots of acid (1M HCl) or base (1M NaOH) to deionized water.

1.2. Synthetic procedures

4,4′-Bis(methyloxycarbonyl)benzil (**1a**) [53], 4,4'-(1,2-dioxo-1,2-ethanediyl)dibenzoic acid [53], 2,3-bis(4-methoxycarbonylphenyl)-6,7-dibromoquinoxaline (**1a**) [38], 2,3-bis(4-methoxycarbonylphenyl)-6,7-bis[(3-aminopropyl)amine]quinoxaline (**QC1**) [38] were prepared according to published procedures.

4,4′-bis(octyloxycarbonyl)benzil (**1b**) was prepared from methyl ester **1a** according a two-step procedure involving sequential hydrolysis and esterification reactions (Scheme S1).

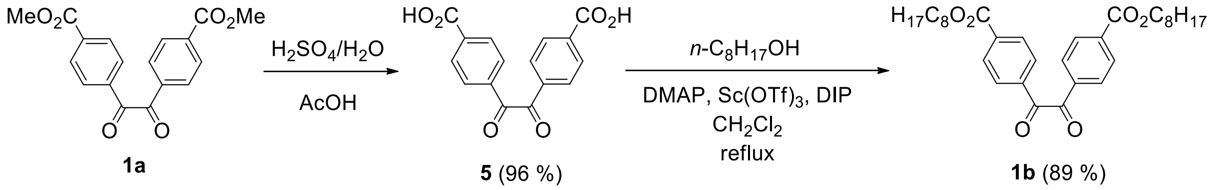

Scheme S1. Synthesis of octyl-substituted diester **1b**.

Acidic hydrolysis of **1a** was performed using published procedure [53].

2,3-Bis(4-methoxycarbonylphenyl)-6,7-dibromoquinoxaline (3a). A 50 mL two-

necked flask equipped with a magnetic stirrer and a back-flow condenser was charged with 4,4′-bis(methyloxycarbonyl)benzil (**1a**) (164 mg, 0.5 mmol) in 50 mL of acetic acid. Subsequently, 4,5-dibromo-1,2-benzenediamine (**2**) (160 mg, 0.6 mmol) was added, and the mixture was stirred at reflux for 24 h. After that, the reaction mixture was cooled to room temperature and stirred for 8 h. The suspension was filtered and dried in vacuo. The target compound (**3a**) was isolated as an orange-brown solid in 89% yield (247 mg). ^{1}H NMR (500 MHz, DMSO-d_6, 25 °C): δ_H = 3.88 (s, 6H, OCH_3), 7.62 (d, J = 8.4 Hz, 4H, H_{Ar}), 7.94 (d, J = 8.4 Hz, 4H, H_{Ar}) and 8.61 (s, 2H, H_Q) ppm. ^{13}C NMR (500 MHz, DMSO-d_6, 25°C): δ_C = 52.59 (2C, OCH_3), 126.77 (2C, $\underline{C}_{Br}$), 129.35 (4C, CH_{Ar}), 130.53 (4C, CH_{Ar}), 130.80 (2C, C_{Ar}), 133.51 (2C, CH_Q), 140.52 (2C, C_N or C_{Ar}), 142.77 (2C, C_N or C_{Ar}), 153.90 (2C, C_N) and 166.24 (2C, $\underline{C}O_2CH_3$) ppm. IR (neat): ν_{max} (cm^{-1}) 3394 (w), 3360 (w), 3339 (w), 3315 (w), 3258 (w), 3081 (w), 2996 (w), 2948 (w), 2903 (w), 2843 (w), 2659 (w), 2360 (w), 2341 (w), 2165 (w), 2148 (w), 2108 (w), 2077 (w), 1981 (w), 1931 (w), 1811 (w), 1718 (s), 1609 (m), 1583 (m), 1572 (m), 1533 (w), 1509 (w), 1436 (m), 1412 (m), 1399 (m), 1336 (m), 1313 (m), 1277 (s), 1188 (m), 1104 (s), 1055 (m), 1018 (m), 980 (m), 964 (m), 931 (m), 898 (m), 859 (m), 828 (m), 770 (m), 730 (m), 722 (m), 706 (s), 693 (m), 647 (m), 639 (m), 616 (m), 605 (m), 551 (m), 517 (m) and 511 (m). HRMS (ESI): *m/z* calcd. for $C_{24}H_{17}Br_2N_2O_4$ $[M + H]^+$ 554.95496, found 554.95470; calcd. for $C_{24}H_{16}Br_2N_2NaO_4$ $[M + Na]^+$ 576.93690, found 576.93612.

2,3-Bis(4-methoxycarbonylphenyl)-6,7-bis[(3-aminopropyl)amino]quinoxaline (QC1). A 250 mL two-necked flask equipped with a magnetic stirrer and a back-flow condenser was charged with 2,3-bis(4-methoxycarbonylphenyl)-6,7-dibromoquinoxaline (**3a**) (2.0 g, 3.597 mmol), dppf (320 mg, 0.576 mmol), Pd(dba)$_2$ (166 mg, 0.288 mmol) and sodium *tert*-butoxide (518 mg, 5.396 mmol). The reaction vessel was evacuated and purged with N_2 three times. Subsequently, 60 mL of dioxane was added with a syringe, and the mixture was stirred. After that, 1,3-diaminopropane (1.8 mL, 21.582 mmol) was added with

a syringe, and the mixture was stirred at reflux for 48 h. Then, the reaction mixture was cooled to room temperature, diluted with CH_2Cl_2 (200 mL), washed with water (2 × 50 mL), dried over $MgSO_4$ and evaporated under reduced pressure. The residue was purified by column chromatography on silica gel using CH_2Cl_2, CH_2Cl_2/MeOH (80:20 v/v), CH_2Cl_2/MeOH (60:40 v/v) and CH_2Cl_2/MeOH/Et_3N (60:40:20 v/v/v) as eluents. The target compound (**QC1**) was isolated as a yellow-orange solid in 25% yield (488 mg). ^1H NMR (300 MHz, $CDCl_3/CD_3OD$ 2:1 v/v, 25 °C): δ_H = 2.16 (br t, 4H, $NHCH_2C\underline{H}_2CH_2NH_2$), 3.12 (br t, 4H, $C\underline{H}_2NH_2$), 3.40 (br t, 4H, $NHC\underline{H}_2$), 3.84 (br s, 6H, OCH_3), 6.83 (s, 2H, H_Q), 7.39 (d, J = 8.2 Hz, 4H, H_{Ar}) and 7.87 (d, J = 8.2 Hz, 4H, H_{Ar}) ppm. NH and NH_2 protons were not unambiguously assigned. ^{13}C NMR (300 MHz, $CDCl_3/CD_3OD$ 2:1 v/v, 25°C): δ_C = 25.59 (2C, $NHCH_2\underline{C}H_2CH_2NH_2$), 37.95 (2C, $\underline{C}H_2NH_2$), 40.48 (2C, $NH\underline{C}H_2$), 52.15 (2C, OCH_3), 101.46 (2C, $\underline{C}NH$), 129.33 (4C, CH_{Ar}), 129.38 (2C, CH_Q), 129.91 (4C, CH_{Ar}), 138.68 (2C, C_{Ar}), 142.18 (2C, C_N or C_{Ar}), 144.00 (2C, C_N or C_{Ar}), 146.52 (2C, C_N) and 167.14 (2C, $\underline{C}O_2CH_3$) ppm. IR (neat): ν_{max} (cm^{-1}) 3381 (w), 3370 (w), 3355 (w), 3327 (w), 3315 (w), 3277 (w), 3261 (w), 3243 (w), 3231 (w), 3219 (w), 3195 (w), 3178 (w), 3157 (w), 3132 (w), 3115 (w), 3060 (w), 3040 (w), 2988 (w), 2961 (w), 2925 (w), 2852 (w), 2812 (w), 2652 (w), 2632 (w), 2531 (w), 2449 (w), 2349 (w), 2324 (w), 2288 (w), 2236 (w), 2221 (w), 2190 (w), 2165 (w), 2144 (w), 2107 (w), 2088 (w), 2064 (w), 2050 (w), 2015 (w), 1981 (w), 1961 (w), 1903 (w), 1842 (w), 1722 (w), 1606 (w), 1505 (w), 1464 (w), 1436 (w), 1404 (w), 1359 (w), 1278 (w), 1211 (w), 1178 (w), 1104 (w), 1019 (w), 982 (w), 865 (w), 781 (m), 631 (m), 536 (m) and 505 (s). HRMS (ESI): *m/z* calcd. for $C_{30}H_{35}N_6O_4$ $[M + H]^+$ 542.27143, found 543.27073.

4,4′-Bis(octyloxycarbonyl)benzil (1b). A 50 mL two-necked flask equipped with a magnetic stirrer and a back-flow condenser was charged with 4,4'-(1,2-dioxo-1,2-ethanediyl)dibenzoic acid (500 mg, 1.68 mmol), DMAP (431 mg, 3.53 mmol) and scandium triflate (83 mg, 0.168 mmol). The reaction vessel was evacuated and purged with N_2 three times. Subsequently, CH_2Cl_2 (10 mL)

and octanol (560 μL, 3.53 mmol) were added by the syringe and the reaction mixture was stirred at room temperature for 30 min. Then N,N'-diisopropylcarbodiimide (552 μL, 3.53 mmol) was added by the syringe and the reaction mixture was stirred at reflux for 24 h. After cooling to room temperature, stirring was continued for 42 h. The suspension was evaporated under reduced pressure with adding of silica gel (2g). The residue was purified by column chromatography on silica gel using ethyl acetate/heptane (gradient elution, from 1:99 v/v to 5:95 v/v) as eluent. The target compound (**1b**) was isolated as a white solid in 89% yield (779 mg).

^{1}H NMR (500 MHz, $CDCl_3$, 25°C): δ_H = 0.90 (t, J = 6.8 Hz, 6H, CH_3), 1.22-1.40 (br m, 16H, $O(CH_2)_3(C\underline{H}_2)_4CH_3$), 1.40-1.52 (m, 4H, $O(CH_2)_2C\underline{H}_2(CH_2)_4CH_3$), 1.74-1.88 (m, 4H, $OCH_2C\underline{H}_2(CH_2)_5CH_3$), 4.37 (t, J = 6.8 Hz, 4H, OCH_2), 8.06 (d, J = 8.4 Hz, 4H, H_{Ar}), 8.19 (d, J = 8.4 Hz, 4H, H_{Ar})) ppm. ^{13}C NMR (500 MHz, $CDCl_3$, 25°C): δ_C = 14.07 (2C, CH_3), 22.62 (2C, CH_2), 25.99 (2C, CH_2), 28.63 (2C, CH_2), 29.16 (2C, CH_2), 29.21 (2C, CH_2), 31.76 (2C, CH_2), 65.87 (2C, OCH_2), 129.82 (4C, CH_{Ar}), 130.10 (4C, CH_{Ar}), 135.69 (2C, C_{Ar}), 135.95 (2C, C_{Ar}), 165.37 (2C, $\underline{C}O_2C_8H_{17}$), 192.98 (2C, $\underline{C}O$) ppm. IR (neat): ν_{max} (cm^{-1}) 2953 (m), 2920 (m), 2885 (m), 2324.12 (w), 1715 (s), 1685 (s), 1671 (s), 1577 (w), 1504 (w), 1467 (m), 1409 (m), 1272 (s), 1208 (s), 1116 (m), 1102 (s), 1013 (m), 939 (m), 900 (m), 858 (w), 826 (w), 782 (w), 723 (s), 686 (m), 569 (m). HRMS (ESI): *m/z* calcd. for $C_{32}H_{43}O_6$ $[M+H]^+$ 523.3054, found 523.3051; calcd. for $C_{32}H_{42}NaO_6$ $[M+Na]^+$ 545.2874, found 545.2872.

2,3-bis(4-octyloxycarbonylphenyl)-6,7-dibromoquinoxaline (3b). A 250 mL two-necked flask equipped with a magnetic stirrer and a back-flow condenser was charged with 4,4′-bis(octyloxycarbonyl)benzil (**1b**) (600 mg, 1.14 mmol) in acetic acid (200 mL). Subsequently, 4,5-dibromo-1,2-benzenediamine (**2**) (366 mg, 1.38 mmol) was added and the mixture was stirred at reflux for 24 h. Then the reaction mixture was cooled to room temperature and stirred for 24 h. The suspension was filtered and the precipitate thus obtained was dissolved in CH_2Cl_2 (50 mL) and dried over $MgSO_4$ for 2 h. Evaporation of this solution under reduced pressure afforded the target compound (**3b**) as a white solid in 84% yield (727 mg). ^{1}H NMR (500 MHz, DMSO-d_6, 25

°C): δ_H = 0.86 (br t, J = 6.7 Hz, 6H, CH_3), 1.13–1.51 (m, 20H, $O(CH_2)_2(C\underline{H}_2)_5CH_3$), 1.62–1.79 (m, 4H, $OCH_2C\underline{H}_2(CH_2)_5CH_3$), 4.29 (t, J = 6.7 Hz, 4H, OCH_2), 7.62 (d, J = 8.1 Hz, 4H, $\underline{Ar}(CO_2C_8H_{17})$), 7.94 (d, J = 8.1 Hz, 4H, $\underline{Ar}(CO_2C_8H_{17})$), 8.61 (s, 2H, Ar(Q)) ppm. ^{13}C NMR (500 MHz, DMSO-d_6, 25 °C): δ_C = 14.17 (2C, CH_3), 22.37 (2C, CH_2), 25.87 (2C, CH_2), 28.59 (2C, CH_2), 28.87 (2C, CH_2), 28.96 (2C, CH_2), 31.55 (2C, CH_2), 65.40 (2C, OCH_2), 126.76 (2C, C_{Br}), 129.29 (4C, CH_{Ar}), 130.51 (4C, CH_{Ar}), 131.07 (2C, C_{Ar}), 133.50 (2C, CH_Q), 140.50 (2C, C_N or C_{Ar}), 142.75 (2C, C_N or C_{Ar}), 153.89 (2C, C_N), 165.75 (2C, CO_2) ppm. IR (neat): ν_{max} (cm^{-1}) 2955 (m), 2925 (m), 2855 (m), 2324 (w), 2288 (w), 2165 (w), 2111 (w) 2051 (w), 2012 (w), 1982 (w), 1940 (w), 1812 (w), 1713 (s, C=O), 1610 (w), 1585 (w), 1572 (w), 1534 (w), 1509 (w), 1466 (m), 1440 (m), 1413 (m), 1389 (m), 1337 (m), 1310 (m), 1269 (s), 1210 (m), 1178 (m), 1101 (s), 1087 (s), 1057 (m), 1016 (s), 981 (m), 954 (m), 897 (m), 881 (m), 865 (m), 860 (m), 810 (w), 776 (m), 724 (m), 708 (s), 694 (m), 638 (w), 627 (m), 615 (m), 605 (m), 548 (m). HRMS (ESI): *m/z* calcd. for $C_{38}H_{45}Br_2N_2O_4$ $[M+H]^+$ 751.17406, found 751.17406.

2,3-bis(4-octyloxycarbonylphenyl)-6,7-bis[(3-aminopropyl)amine]quinoxaline (QC8). A 10 mL two-necked flask equipped with a magnetic stirrer and a back-flow condenser was charged with 2,3-bis(4-octyloxycarbonylphenyl)-6,7-dibromoquinoxaline (**3b**) (100 mg, 0.132 mmol), dppf (11.6 mg, 0.021 mmol), $Pd(dba)_2$ (6.3 mg, 0.011 mmol) and sodium *tert*-butoxide (19 mg, 0.198 mmol). The reaction vessel was evacuated and purged with N_2 three times. Subsequently, 4 mL of dioxane was added by the syringe and the mixture was stirred. After that 66.5 µL of 1,3-diaminopropane (0.799 mmol) was added by the syringe and the mixture was stirred at reflux for 48 h. The mixture was washed by water and dried over $MgSO_4$ for 2 h and evaporated under reduced pressure. The residue was purified by column chromatography on silica gel using CH_2Cl_2, CH_2Cl_2/MeOH (4/1 v/v), CH_2Cl_2/MeOH (3/2 v/v) and CH_2Cl_2/MeOH/Et_3N (3/3/1 v/v/v) as eluents. The target compound (**QC8**) was isolated as yellow solid in 24 % yield (21 mg). ^1H NMR (500 MHz, $CDCl_3/CD_3OD$ 2:1 v/v, 25 °C): δ_H = 0.80 (t, J = 6.8 Hz, 6H, CH_3), 1.11–1.42 (br m, 20H, $OCH_2CH_2(C\underline{H}_2)_5CH_3$), 1.63–1.79 (m, 4H, $OCH_2C\underline{H}_2(CH_2)_5CH_3$), 2.16 (br t, 4H,

$C\underline{H}_2CH_2NH_2$), 3.12 (br t, 4H, $C\underline{H}_2NH_2$), 3.40 (br t, 4H, $NHC\underline{H}_2$), 4.23 (t, J = 6.7 Hz, 4H, OCH_2), 6.82 (br s, 2H, H_Q), 7.40 (d, J = 8.5 Hz, 4H, H_{Ar}), 7.88 (d, J = 8.5 Hz, 4H, H_{Ar}) ppm. NH and NH_2 protons were not unambiguously assigned. ^{13}C NMR (500 MHz, $CDCl_3/CD_3OD$ 2:1 v/v, 25°C): δ_C = 13.76 (2C, CH_3), 22.49 (2C, CH_2), 25.54 (2C, $NHCH_2\underline{C}H_2CH_2NH_2$ or CH_2), 25.93 (2C, $NHCH_2\underline{C}H_2CH_2NH_2$ or CH_2), 28.54 (2C, CH_2), 29.05 (2C, CH_2), 29.11 (2C, CH_2), 31.67 (2C, CH_2), 37.84 (2C, CH_2), 40.44 (2C, CH_2NH_2), 53.39 (2C, $NH\underline{C}H_2$), 65.43 (2C, OCH_2), 101.32 (2C, $\underline{C}NH$), 129.33 (4C, CH_{Ar}), 129.71 (2C, CH_Q), 129.87 (4C, CH_{Ar}), 138.69 (2C, C_{Ar}), 142.23 (2C, C_N or C_{Ar}), 143.95 (2C, C_N or C_{Ar}), 146.56 (2C, C_N), 166.75 (2C, CO_2) ppm. IR (neat): λ_{max} (cm^{-1}) 3382 (w), 3370 (w), 3355 (w), 3335 (w), 3324 (w), 3310 (w), 3299 (w), 3278 (w), 3261 (w), 3230 (w), 3221 (w), 3196 (w), 3187 (w), 3177 (w), 3167 (w), 3142 (w), 3115 (w), 3098 (w), 3083 (w), 3041 (w), 3005 (w), 2957 (w), 2926 (w), 2855 (w), 2662 (w), 2596 (w), 2584 (w), 2511 (w), 2454 (w), 2410 (w), 2394 (w), 2349 (w), 2324 (w), 2288 (w), 2179 (w), 2169 (w), 2137 (w), 2100 (w), 2051 (w), 2039 (w), 2016 (w), 1981 (w), 1962 (w), 1933 (w), 1902 (w), 1721 (w), 1682 (w), 1651 (w), 1610 (w), 1515 (w), 1465 (w), 1405 (w), 1379 (w), 1310 (w), 1274 (w), 1178 (w), 1106 (w), 1019 (w), 989 (w), 893 (w), 862 (w), 779 (m), 710 (m), 631 (m), 533 (s). HRMS (ESI): *m/z* calcd. for $C_{44}H_{63}N_6O_4$ $[M+H]^+$ 739.4905, found 739.4905.

2. NMR spectra for compounds

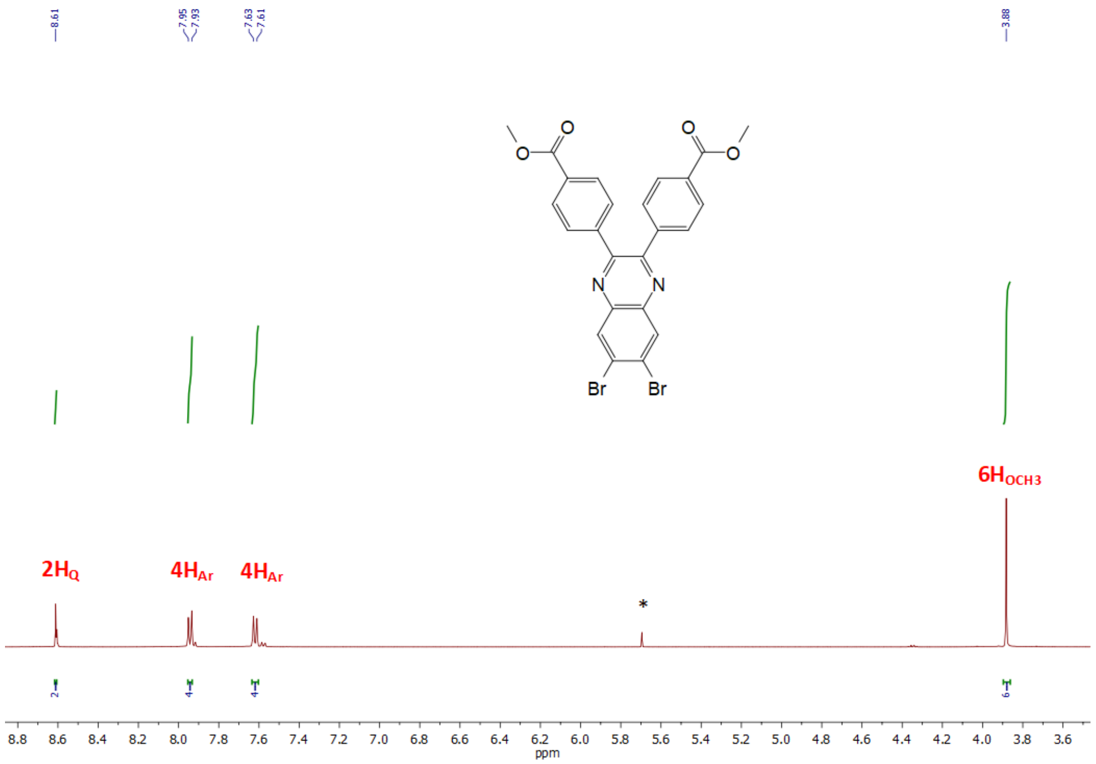

Figure S1. ^{1}H NMR spectrum of **3a** (DMSO-d_6). Peak of CH_2Cl_2 (δ_H 5.69 ppm) is indicated with a star.

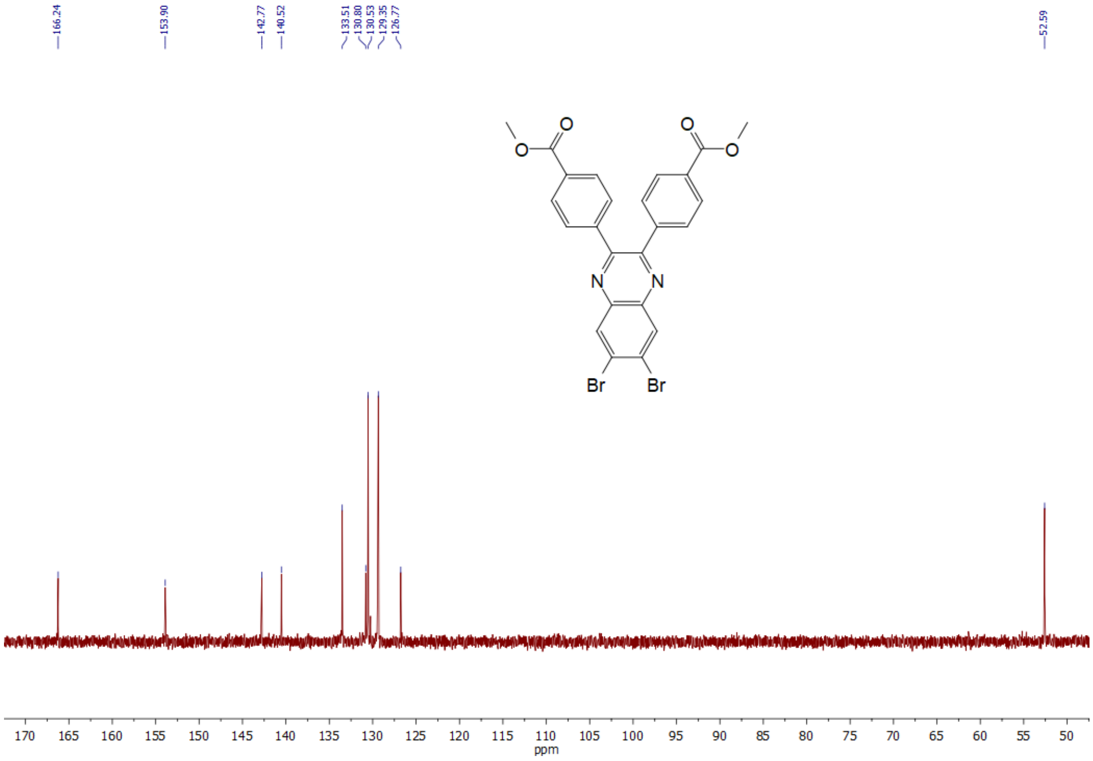

Figure S2. ^{13}C NMR spectrum of **3a** (DMSO-d_6).

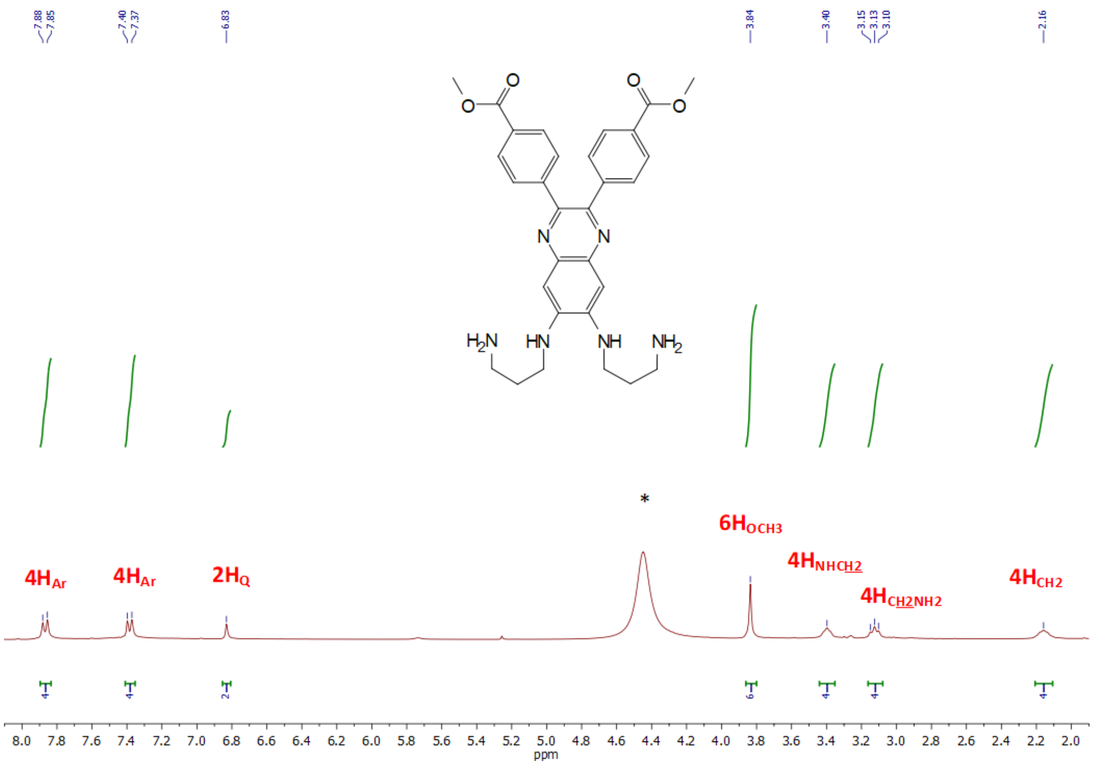

Figure S3. ^{1}H NMR spectrum of **QC1** ($CDCl_3$/CD_3OD 2:1, v/v). Peak of water (δ_H 4.45 ppm) is indicated with a star. Signals are broadened probably due to a partial protonation of the compound.

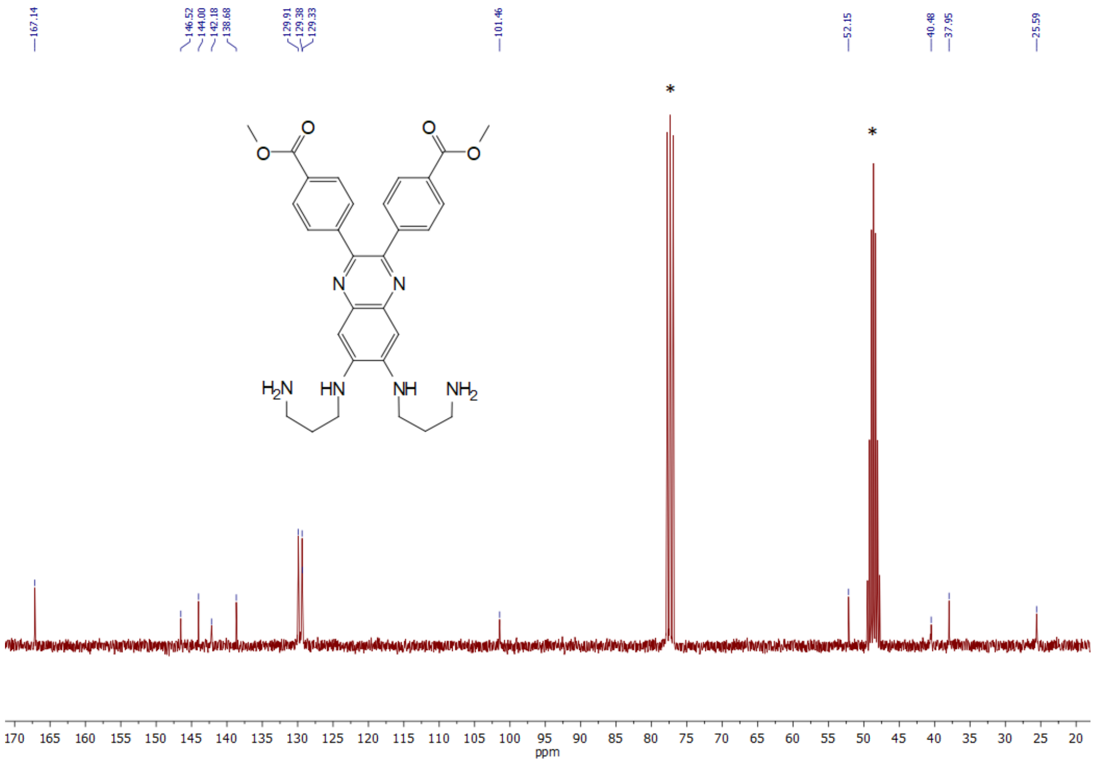

Figure S4. ^{13}C NMR spectrum of **QC1** ($CDCl_3$/CD_3OD 2:1, v/v). Residual peaks of solvents (δ_C 77.32 ppm – $CDCl_3$, δ_C 48.64 ppm – MeOD) are indicated with stars.

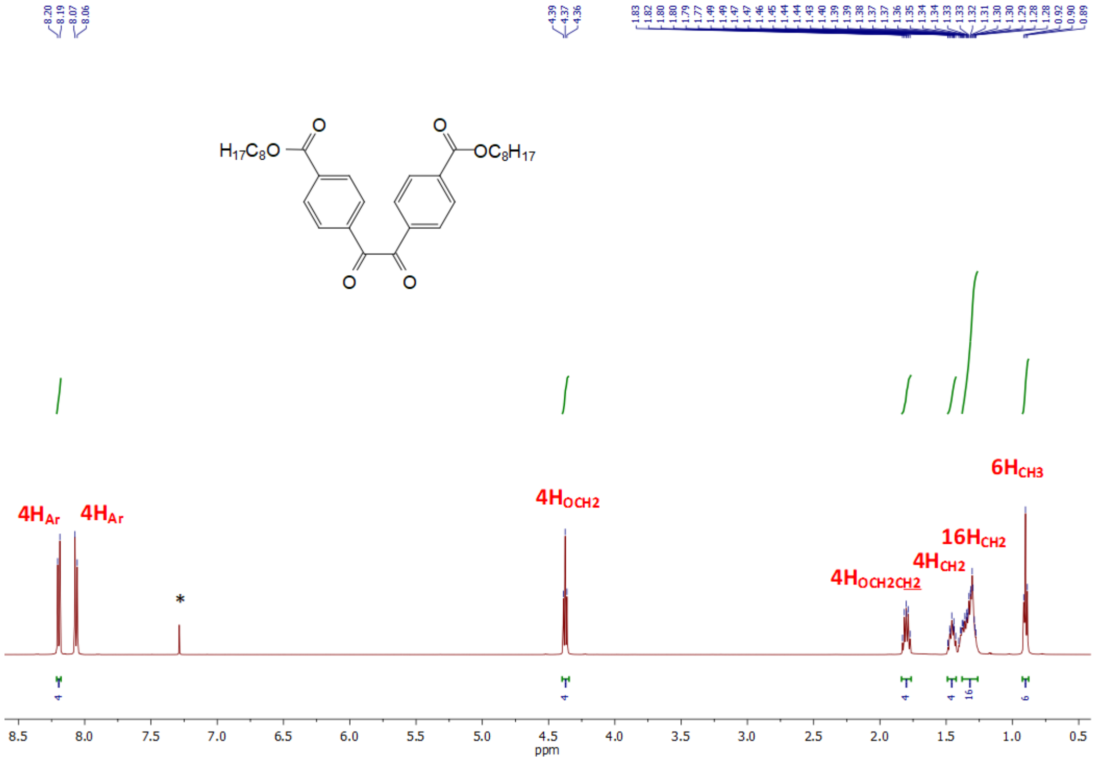

Figure S5. ^{1}H NMR spectrum of **1b** ($CDCl_3$). Residual peak of $CHCl_3$ (δ_H 7.29 ppm) is indicated with a star.

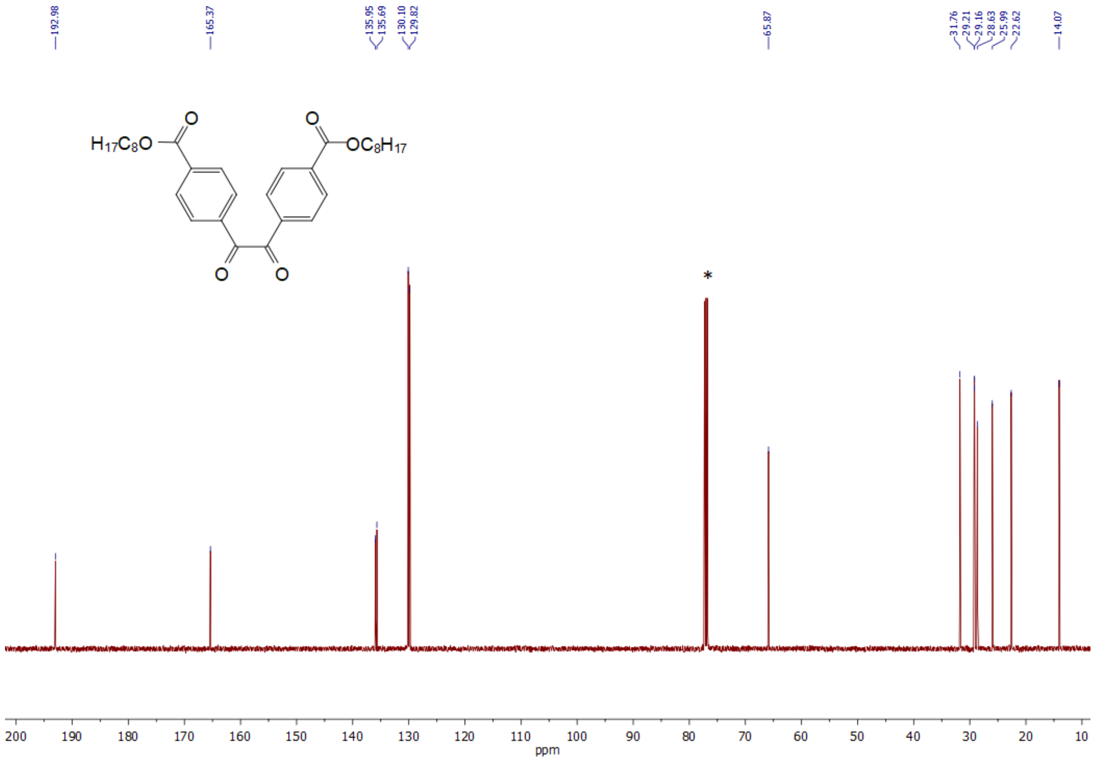

Figure S6. ^{13}C NMR spectrum of **1b** ($CDCl_3$). A residual peak of $CHCl_3$ (δ_C 77.02 ppm) is indicated with a star.

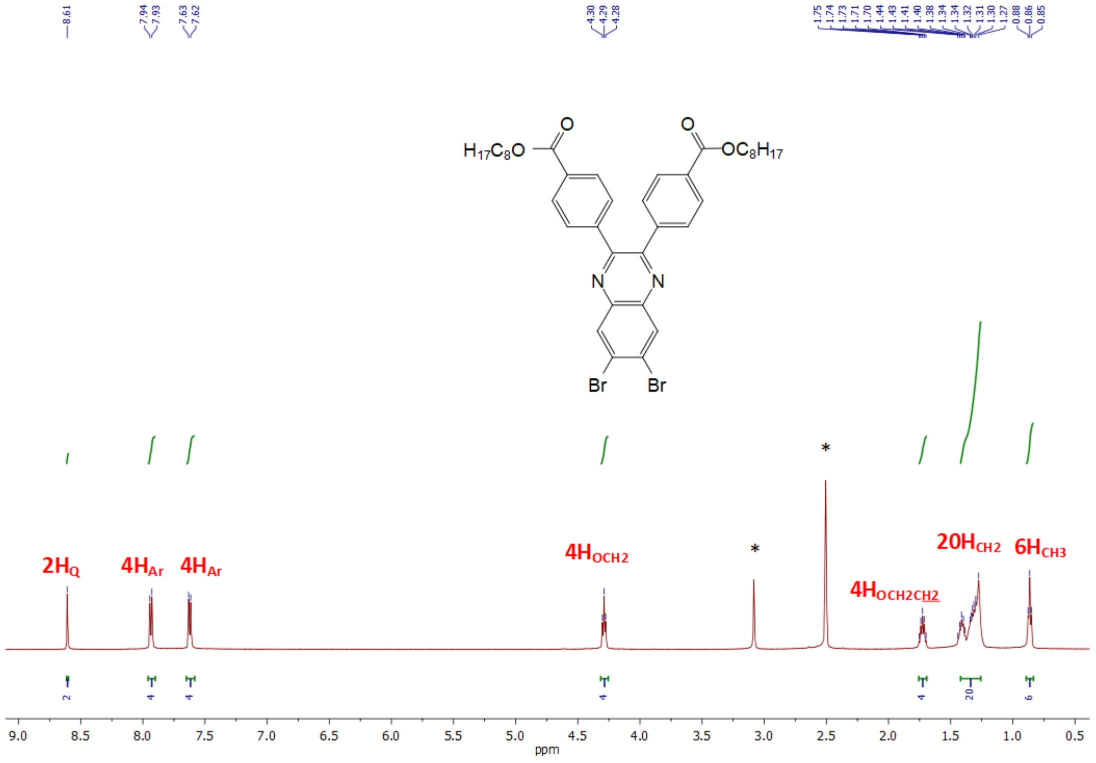

Figure S7. ^{1}H NMR spectrum of **3b** (DMSO-d_6). Residual peak of DMSO (δ_H 2.51 ppm) and a peak of water (δ_H 3.09 ppm) are indicated with stars.

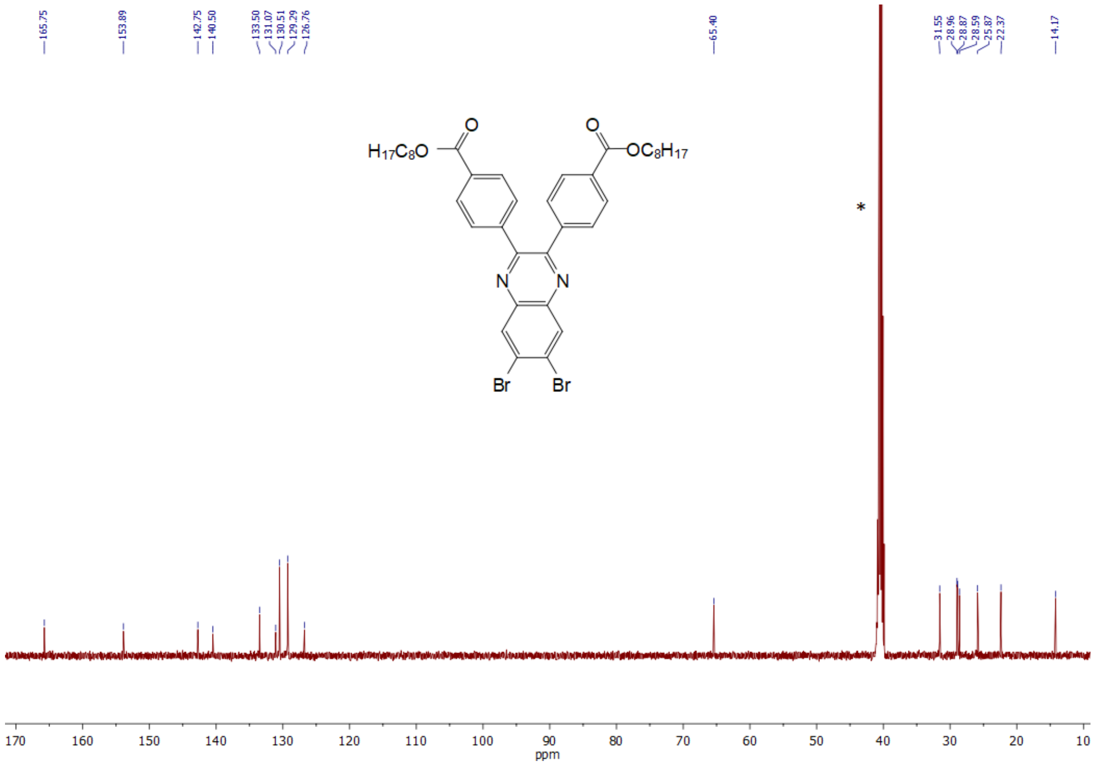

Figure S8. ^{13}C NMR spectrum of **3b** (DMSO-d_6). Residual peak of solvent (δ_C 40.55 ppm) is indicated with a star.

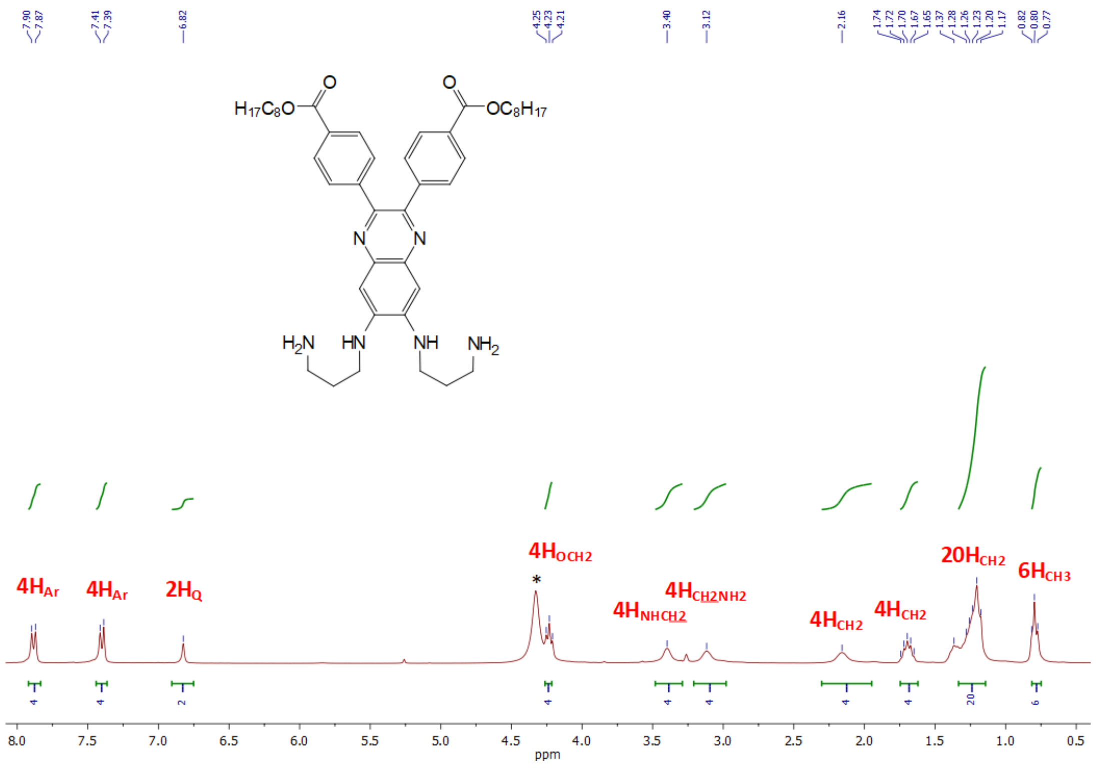

Figure S9. ^{1}H NMR spectrum of **QC8** ($CDCl_3/CD_3OD$ 2:1, v/v). Peak of water (δ_H 4.32 ppm) is indicated with a star. Signals are broadened probably due to a partial protonation of compound.

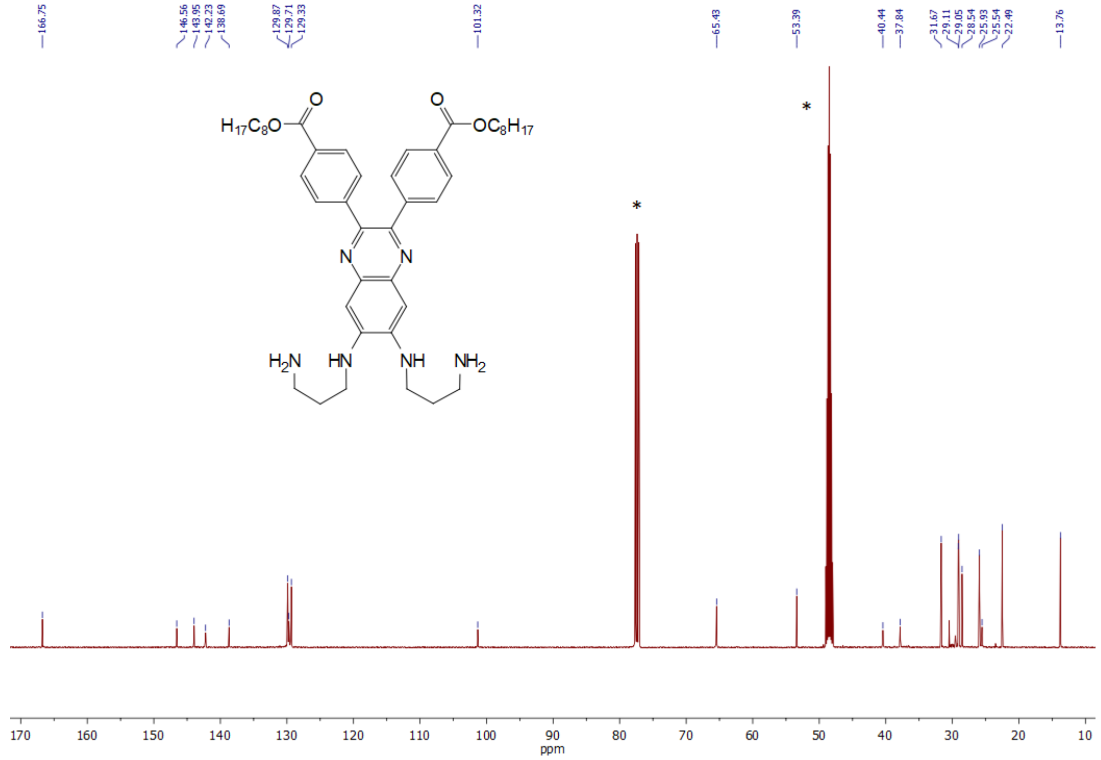

Figure S10. ^{13}C NMR spectrum of **QC8** ($CDCl_3/CD_3OD$ 2:1, v/v). Residual solvent peaks (δ_C 77.32 ppm – $CDCl_3$, δ_C 48.64 ppm – MeOD) are indicated with stars.

3. HRMS (ESI) spectra for compounds

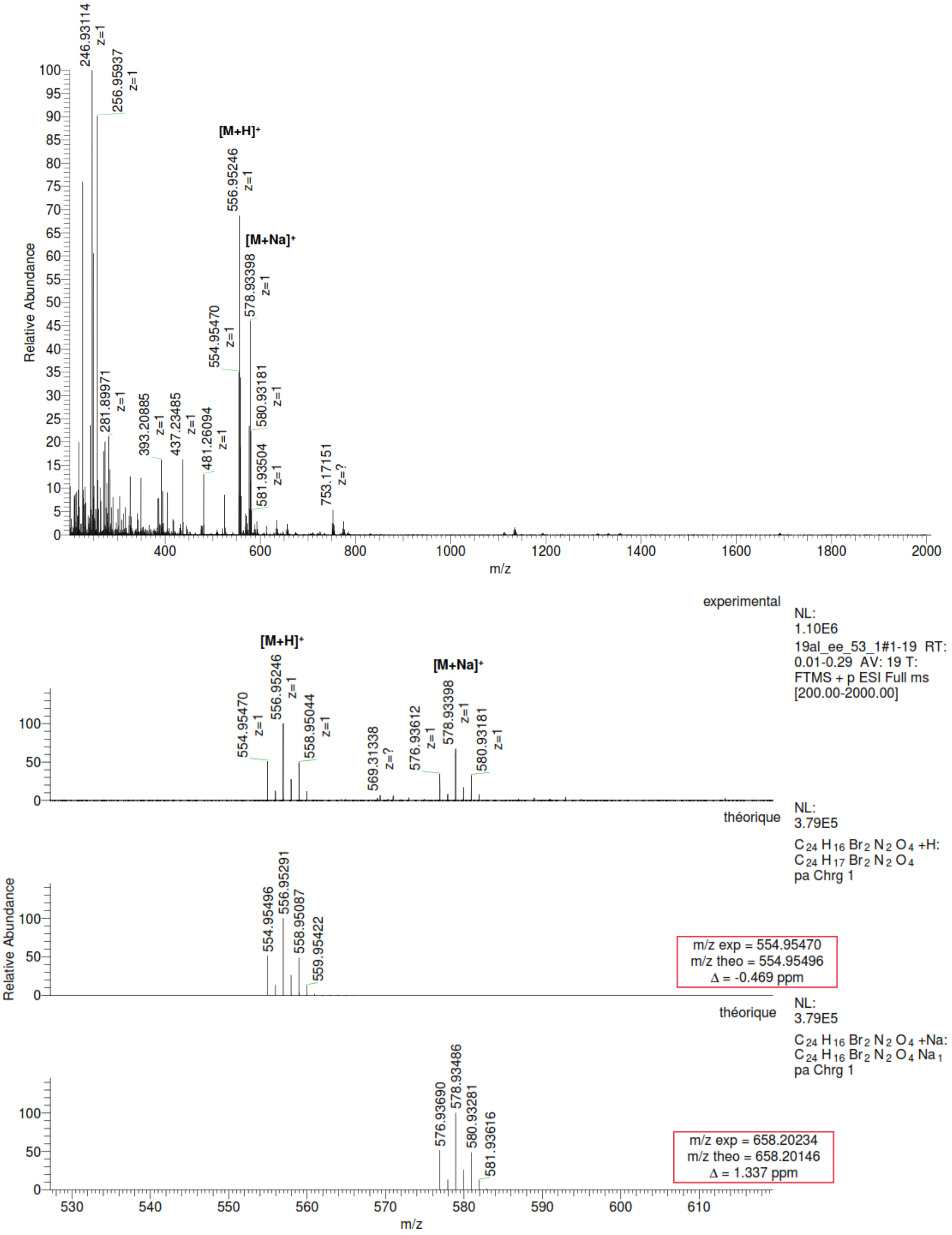

Figure S11. HR-ESI mass spectrum of **3a**.

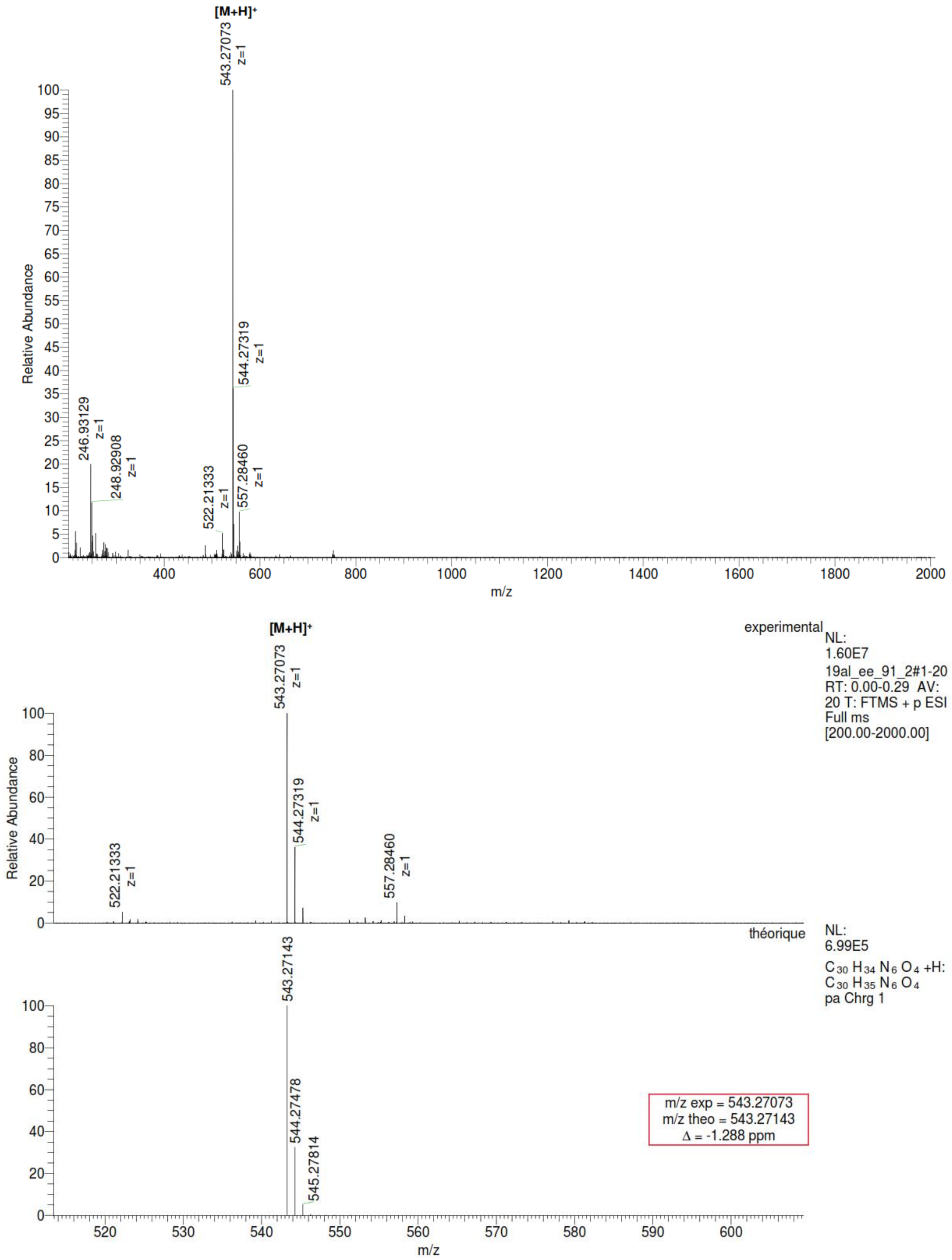

Figure S12. HR-ESI mass spectrum of **QC1**.

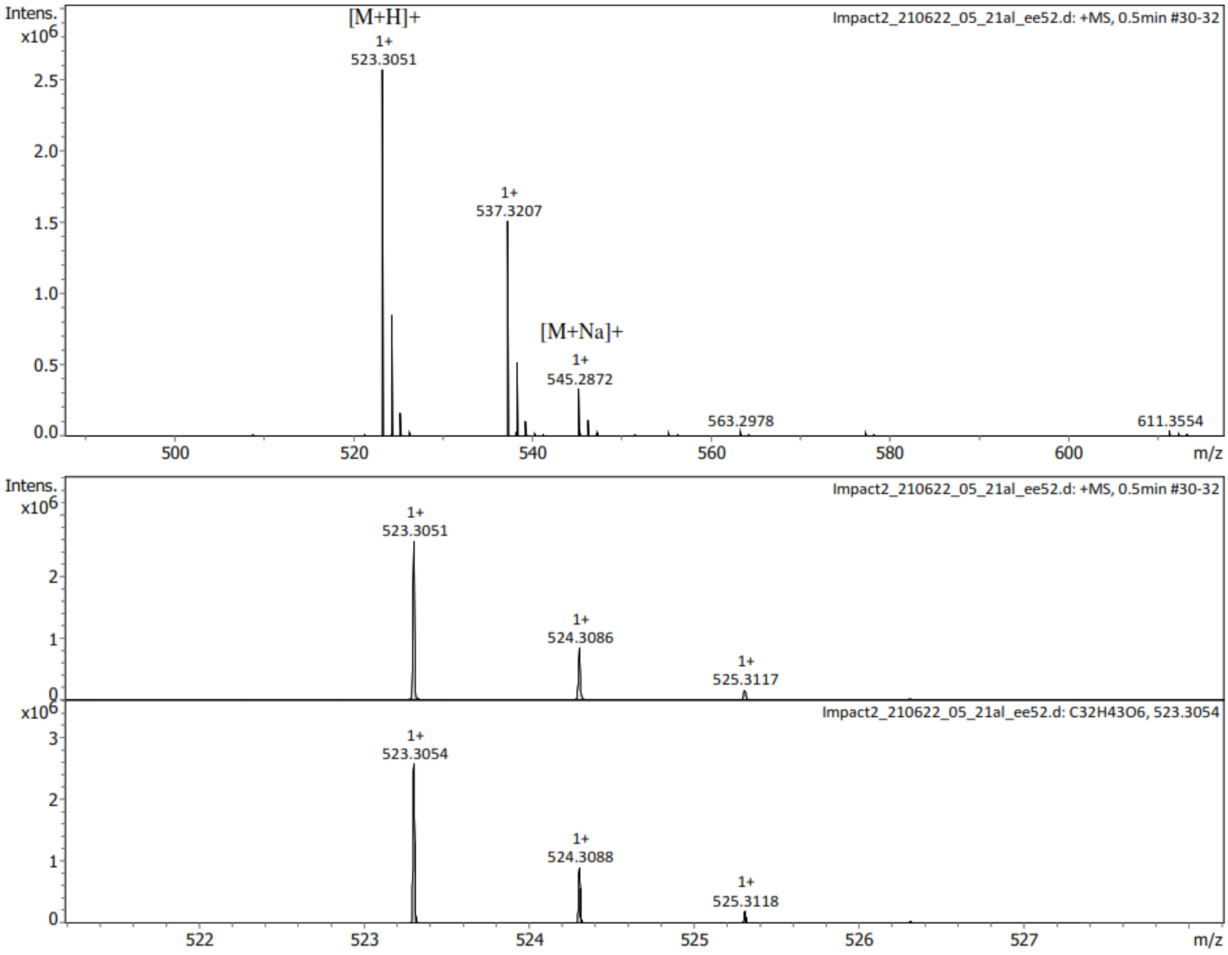

Meas. m/z	Ion Formula	m/z	Sum Formula	err [ppm]	mSigma	Adduct	z
523.3051	C32H43O6	523.3054	C32H42O6	0.7	11.8	M+H	1+
545.2872	C32H42NaO6	545.2874		0.3	2.2	M+Na	1+

Figure S13. HR-ESI mass spectrum of **1b**.

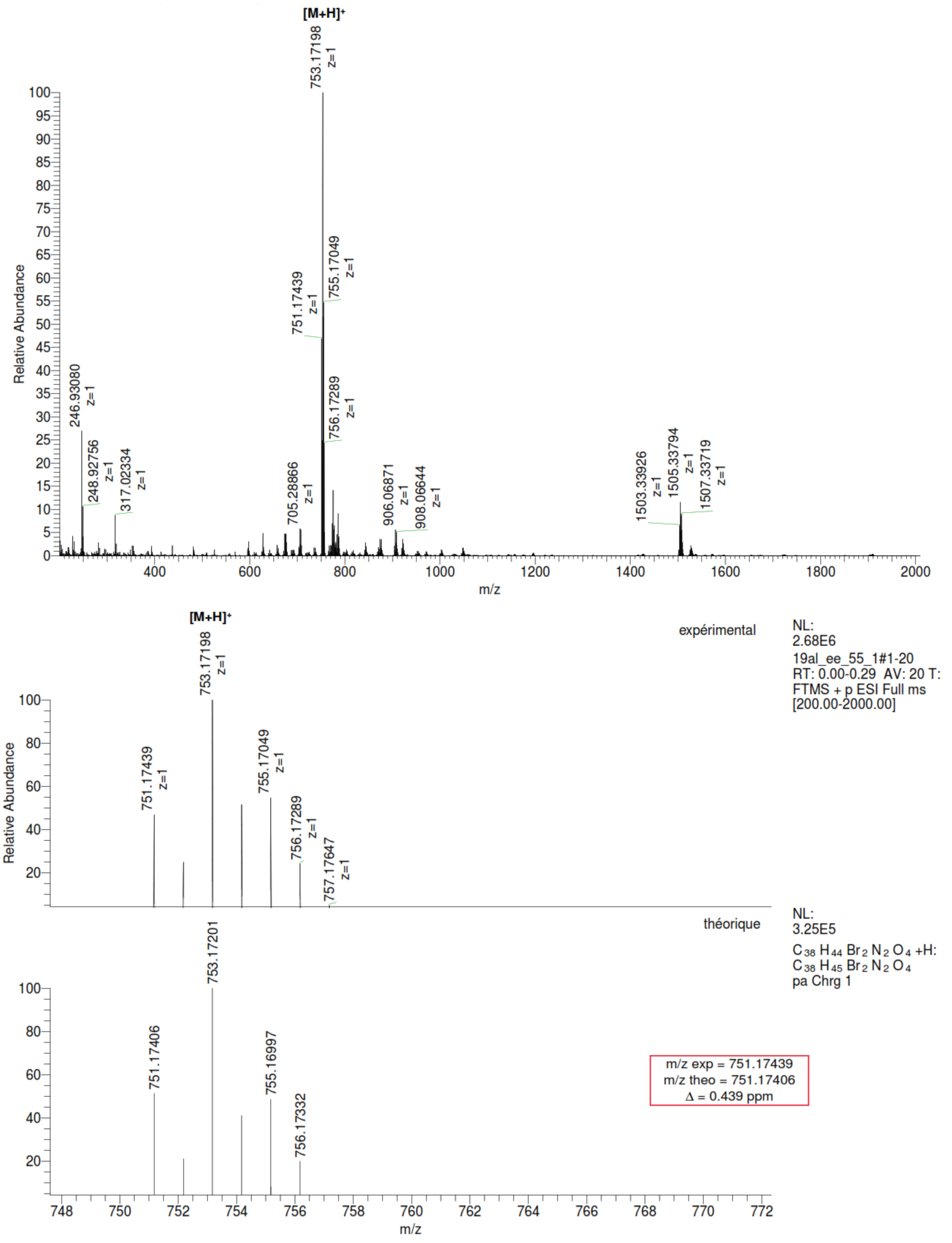

Figure S14. HR-ESI mass spectrum of **3b**.

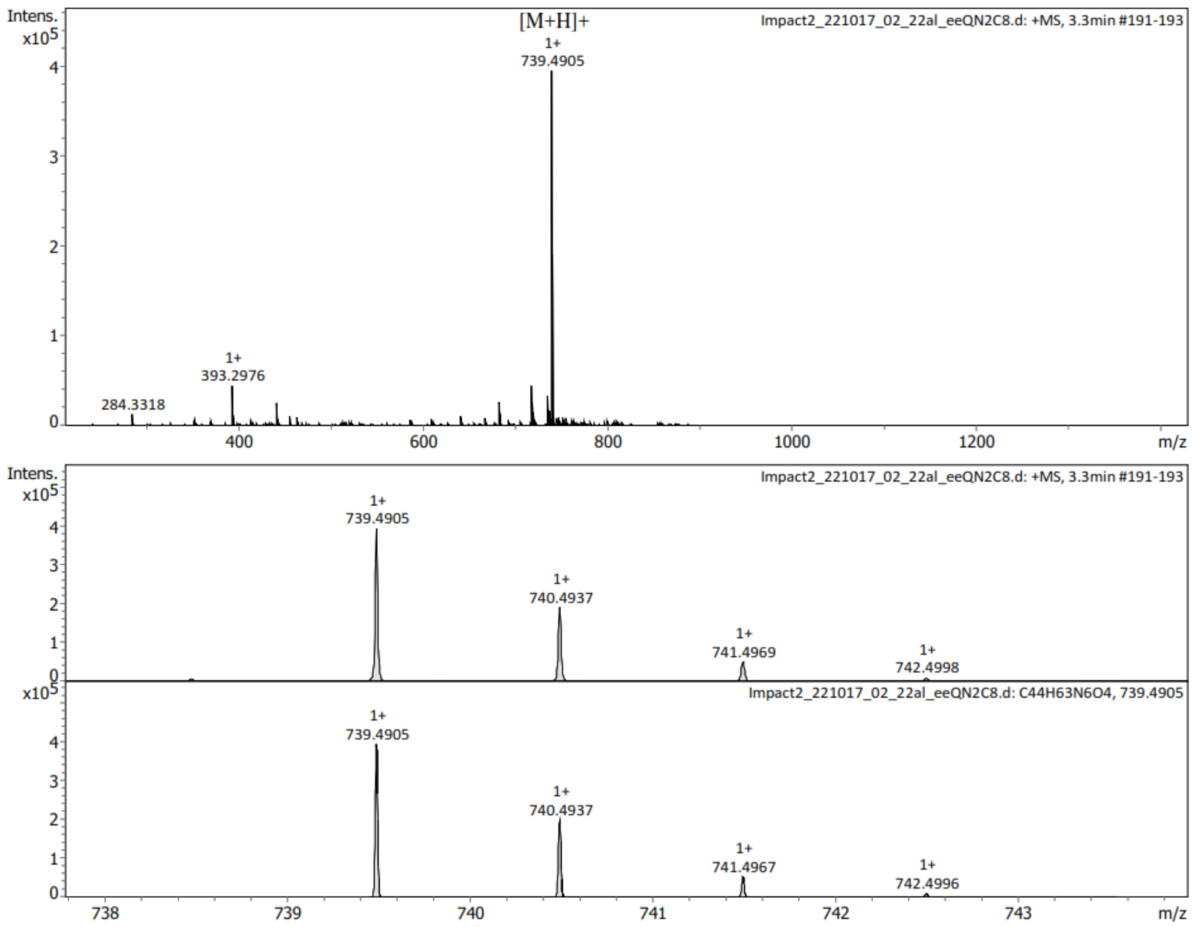

Meas. m/z	Ion Formula	m/z	Sum Formula	err [ppm]	mSigma	Adduct	z
739.4905	C44H63N6O4	739.4905	C44H62N6O4	0.1	12.5	M+H	1+

Figure S15. HR-ESI mass spectrum of **QC8**.

4. Spectroscopic studies of QC1 and QC8 in solutions

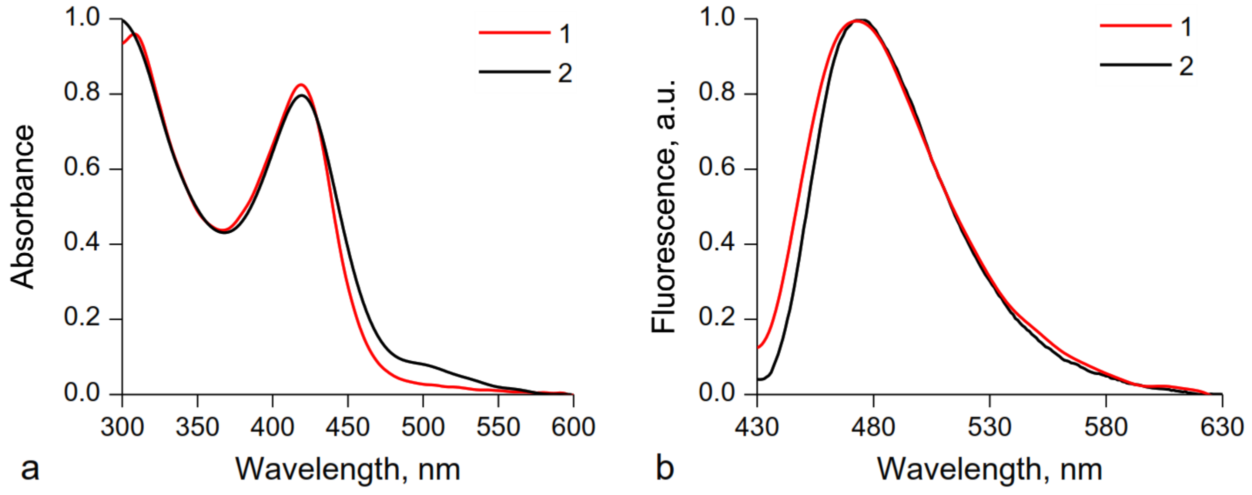

Figure S16. Normalized [0; 1] UV–vis absorption (a) and fluorescence (b) spectra of **QC1** (*1*) and **QC8** (*2*) in chloroform solution, c = 0.1 mM, λ_{ex} = 420 nm.

Table S1. Photophysical data for aminoquinoxalines **QC1** and **QC8** in chloroform solution.

Ligand	λ_{abs}, nm (log (ε, cm^{-1} M^{-1}))	λ_{em}, nm[1]	Φ_{em}[2]
**QC1**	308 (3.76), 420 (3.69)	472	0.244
**QC8**	300 (3.79), 420 (3.70)	474	0.103

[1] Emission was excited at 420 nm. [2] Measured in chloroform at ambient temperatures relative to a solution of fluorescein in ethanol as a standard.

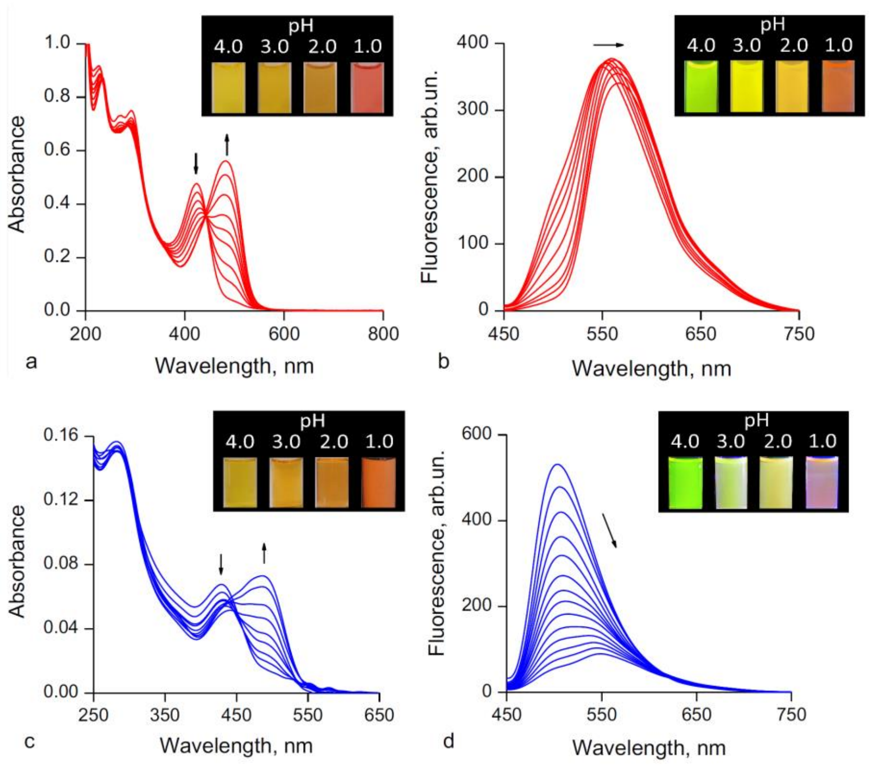

Figure S17. UV–vis absorption (a, c) and fluorescence (b, d) spectra of **QC1** (a, b) in an aqueous solution and **QC8** (b, d) in water-methanol (1:1, v/v) solution upon pH titration (pH = 4.0 – 1.0). [**QC1**] = 0.112 mM (a) and 0.015 mM (b), [**QC8**] = 0.015 mM (b, d); λ_{ex} = 420 nm. Inserts: pH-induced color changes of solutions in daylight (a, c) and under irradiation by LED (λ = 365 nm) (b, d).

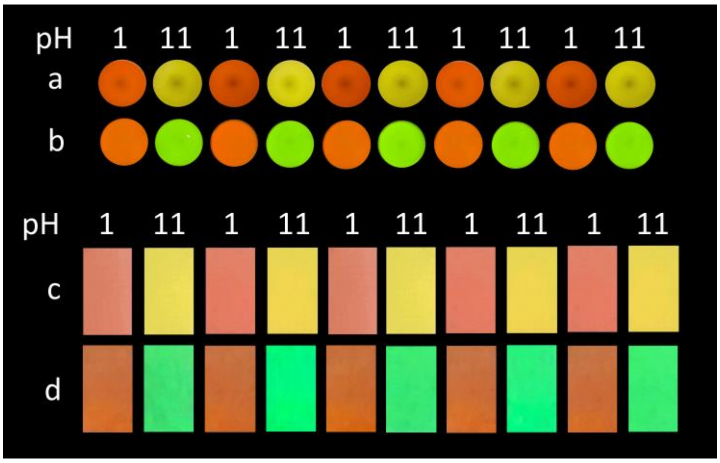

Figure S18. pH-induced color changes of polymer films (a, b) and test strips (c, d) based on **QC1** in (a, c) daylight and (b, d) under irradiation by LED (λ = 365 nm) upon sequential exposure in aqueous solutions with pH 1 and 11 (recycling experiments).

5. Monolayer of QC8 at the air–water interface

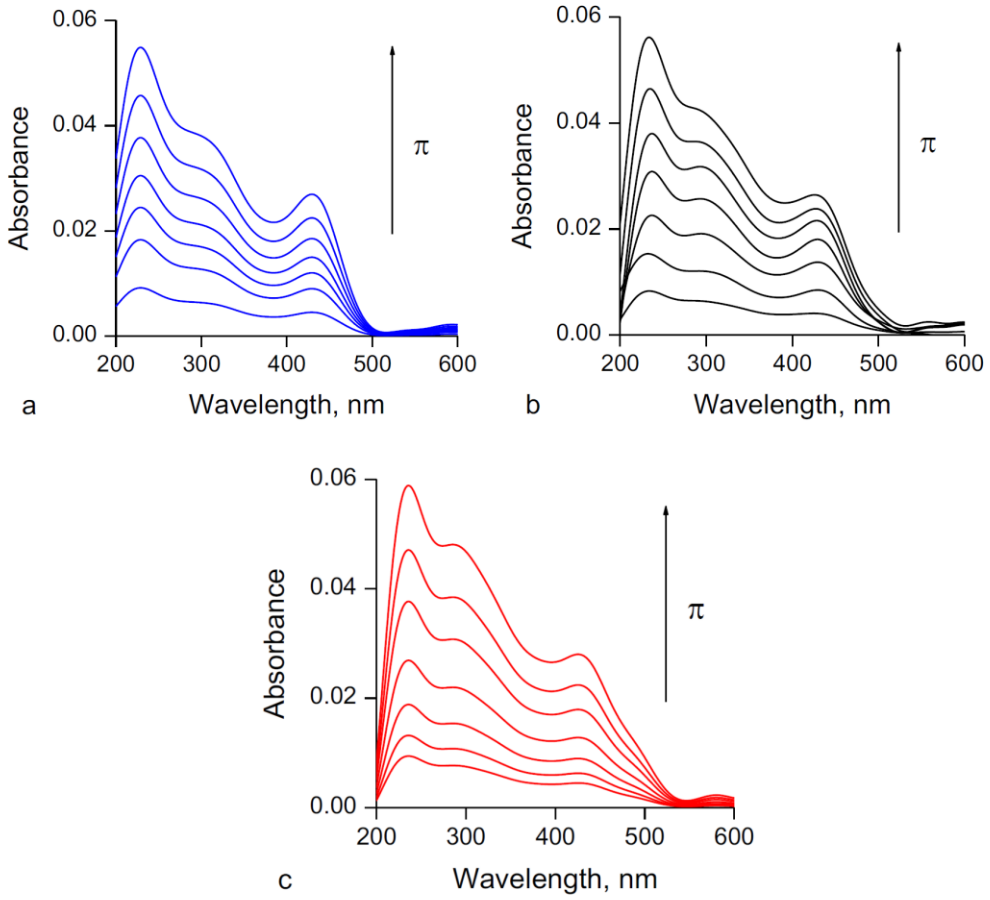

Figure S19. *In situ* UV–vis absorption spectra for the monolayer of **QC8** on the pure water surface at pH (a) 11.0, (b) 5.5 and (c) 1.0 under compression.

6. LB and LS films of QC8

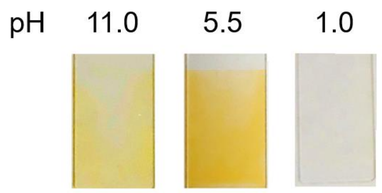

Figure S20. Photographs of 30-layer **QC8** LB films transferred onto quartz substrate at 20 mN m^{-1} from surface of pure water subphase with pH 11.0, 5.5 and 1.0.

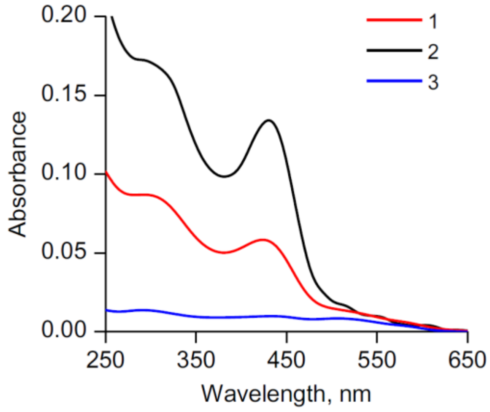

Figure S21. UV–vis absorbance spectra of 30-layer LB film of **QC8** transferred onto quartz substrate at 20 mN m^{-1} from surface of pure water subphase with pH 11.0 (*1*), 5.5 (*2*) and 1.0 (*3*).

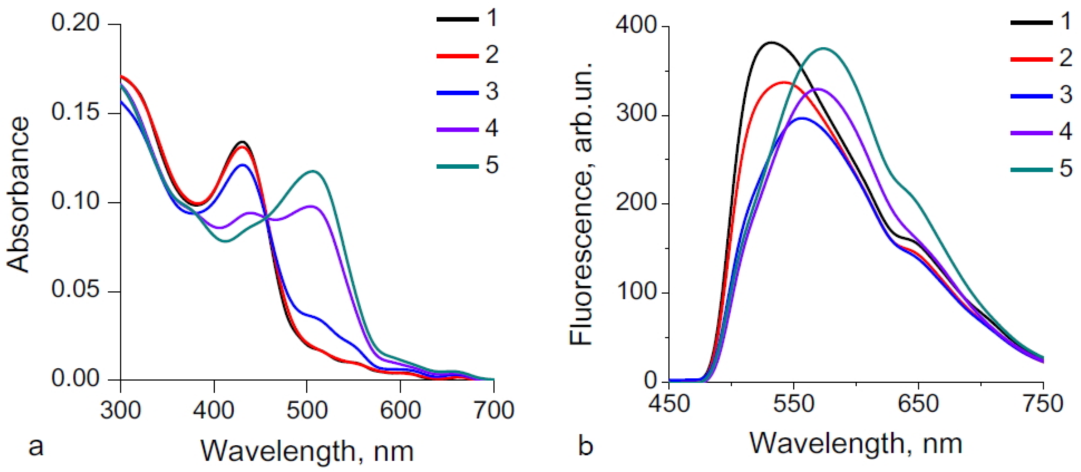

Figure S22. UV–vis absorption (a) and fluorescence (b) spectra of 30-layer LB film of **QC8** (*1*) transferred onto quartz substrate, at 20 mN m^{-1} and immersed in aqueous solutions with pH 3.0 (*2*), 2.0 (*3*), 1.5 (*4*) and 1.0 (*5*) for 30 s, λ_{ex} = 420 nm.

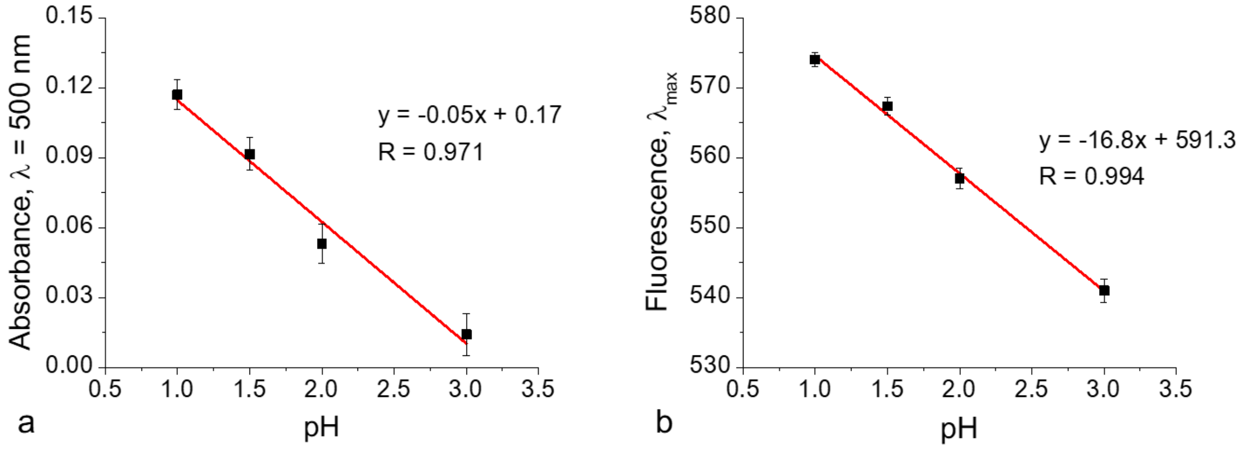

Figure S23. Dependence of the absorbance intensity (a) and fluorescence λ_{max} (b) on pH value for 30-layer LB film of **QC8** transferred onto PVC substrate at 20 mN m^{-1}.

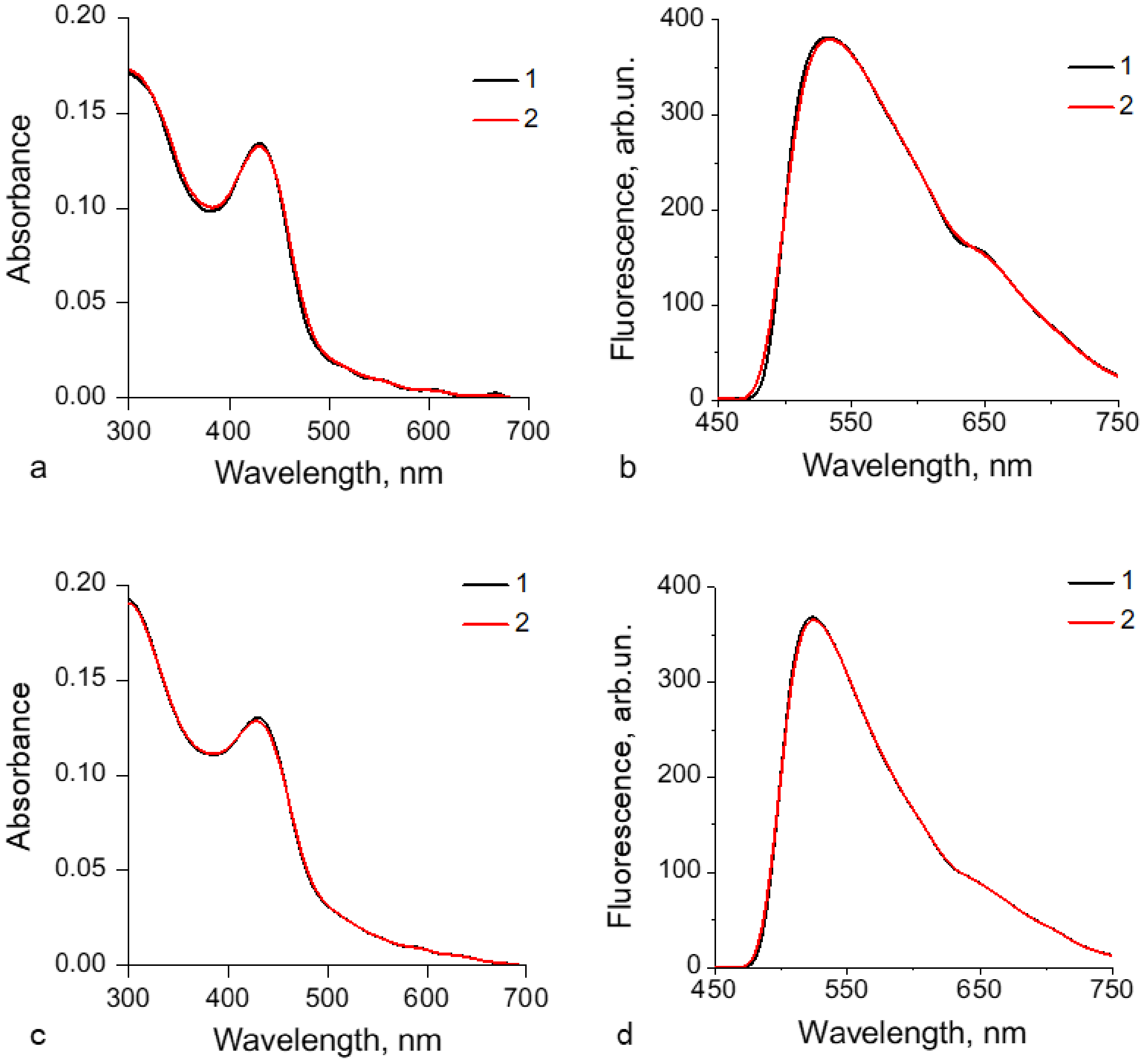

Figure S24. UV–vis absorption (a, c) and fluorescence (b, d) spectra of 30-layer LB (a, b) and LS (c, d) films of **QC8** immersed in deionized water acidified by HCl to pH 4.52 (*1*) and in 0.1 mM aqueous solution of metal cations (K^+, Na^+, Mg^{2+}, Ba^{2+}, Ca^{2+}, Zn^{2+}, Co^{2+}, Cd^{2+}, Pb^{2+}, Ag^+, Ni^{2+}, Hg^{2+}, Cu^{2+}, Al^{3+}; pH = 4.52) for 10 minutes.

7. Halochromic materials for qualitative pH measurement in the range of 1–4

Table S2. Halochromic materials for qualitative pH measurements in the pH range of 1–4.

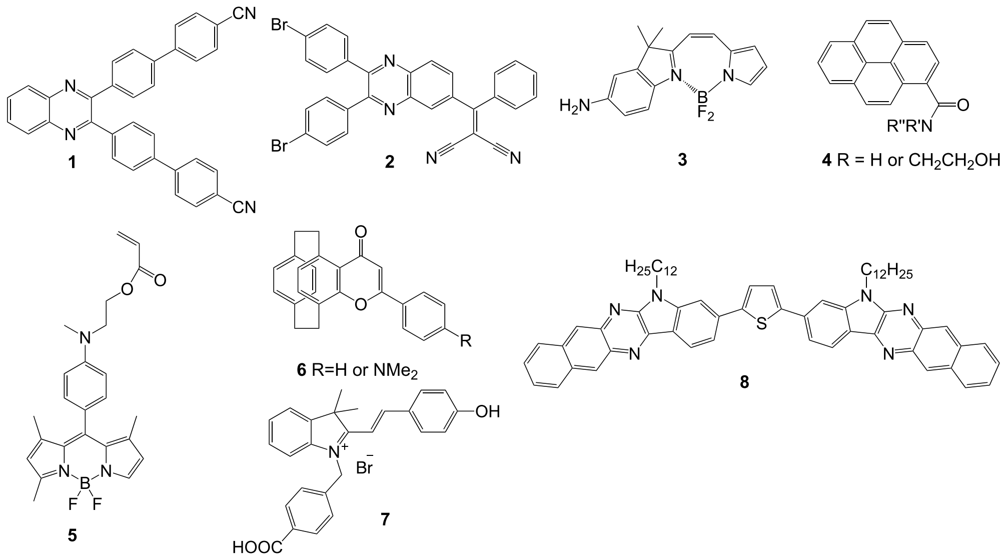

Entry	Dye	Support	Immobilization technique	pH range or specific acidic analyte (Response time)	Signal registration	Accuracy (qualitative/ quantitative)	Stability and reusability	Ref.
1	1	Paper strips	Impregnation	TFA	**colorimetry[1] (day light, LED[2])** fluorescence	qualitative	n/d[3]	[24]
2	2	Paper strips	Impregnation	TFA	colorimetry (day light)	qualitative	was recovered	[22]
3	3	Paper strips	Impregnation	HCl(vapor)	colorimetry (LED)	qualitative	was recovered	[43]
4	4	Paper strips	Impregnation	pH = 1–3	colorimetry (LED)	qualitative	n/d	[44]
5	5	Paper strips	Impregnation	HCl(vapor)	colorimetry (LED)	qualitative	was reused 5 times (recovering takes 5 s)	[50]
6	6	Paper strips	Impregnation	TFA (immediately)	**colorimetry (day light, LED[2])** spectrophotometry fluorescence	qualitative	was reused 10 times	[45]
7	7	Paper strips	Impregnation	HCl	**colorimetry (day light, LED[4])**	qualitative	was reused 5 times	[48]
8	8	Solid substrate	Drop-casting	HCl (immediately)	**colorimetry (day light, LED[2])** spectrophotometry fluorescence	qualitative	n/d	[42]

[1] Dual-responsive materials are marked in bold. [2] Illumination at 365 nm. [3] n/d – not determined. [4] Illumination at UV light.
